# Application of simulation-based CYP26 SNP-environment barcodes for evaluating the occurrence of oral malignant disorders by odds ratio-based binary particle swarm optimization: A case-control study in the Taiwanese population

**DOI:** 10.1371/journal.pone.0220719

**Published:** 2019-08-29

**Authors:** Ping-Ho Chen, Li-Yeh Chuang, Kuo-Chuan Wu, Yan-Hsiung Wang, Tien-Yu Shieh, Jim Jinn-Chyuan Sheu, Hsueh-Wei Chang, Cheng-Hong Yang

**Affiliations:** 1 School of Dentistry, College of Dental Medicine, Kaohsiung Medical University, Kaohsiung, Taiwan; 2 Center for Cancer Research, Kaohsiung Medical University, Kaohsiung, Taiwan; 3 Institute of Biomedical Sciences, National Sun Yat-sen University, Kaohsiung, Taiwan; 4 Department of Chemical Engineering, I-Shou University, Kaohsiung, Taiwan; 5 Institute of Biotechnology and Chemical Engineering, I-Shou University, Kaohsiung, Taiwan; 6 Department of Electronic Engineering, National Kaohsiung University of Science and Technology, Kaohsiung, Taiwan; 7 Department of Computer Science and Information Engineering, National Kaohsiung University of Sciences and Technology, Kaohsiung, Taiwan; 8 Orthopaedic Research Center, Kaohsiung Medical University, Kaohsiung, Taiwan; 9 Global Center of Excellence for Oral Health Research and Development, Kaohsiung, Taiwan; 10 Institute of Biopharmaceutical Sciences, National Sun Yat-sen University, Kaohsiung, Taiwan; 11 School of Chinese Medicine, China Medical University, Taichung, Taiwan; 12 Department of Health and Nutrition Biotechnology, Asia University, Taichung, Taiwan; 13 Institute of Medical Science and Technology, National Sun Yat-sen University, Kaohsiung, Taiwan; 14 Department of Medical Research, Kaohsiung Medical University Hospital, Kaohsiung, Taiwan; 15 Department of Biomedical Science and Environmental Biology, Kaohsiung Medical University, Kaohsiung, Taiwan; 16 Ph. D. Program in Biomedical Engineering, Kaohsiung Medical University, Kaohsiung, Taiwan; Shantou University, CHINA

## Abstract

**Introduction:**

Genetic polymorphisms and social factors (alcohol consumption, betel quid (BQ) usage, and cigarette consumption), both separately or jointly, play a crucial role in the occurrence of oral malignant disorders such as oral and pharyngeal cancers and oral potentially malignant disorders (OPMD).

**Material and methods:**

Simultaneous analyses of multiple single nucleotide polymorphisms (SNPs) and environmental effects on oral malignant disorders are essential to examine, albeit challenging. Thus, we conducted a case-control study (N = 576) to analyze the risk of occurrence of oral malignant disorders by using binary particle swarm optimization (BPSO) with an odds ratio (OR)-based method.

**Results:**

We demonstrated that a combination of SNPs (*CYP26B1* rs887844 and *CYP26C1* rs12256889) and socio-demographic factors (age, ethnicity, and BQ chewing), referred to as the combined effects of SNP-environment, correlated with maximal risk diversity of occurrence observed between the oral malignant disorder group and the control group. The risks were more prominent in the oral and pharyngeal cancers group (OR = 10.30; 95% confidence interval (CI) = 4.58–23.15) than in the OPMD group (OR = 5.42; 95% CI = 1.94–15.12).

**Conclusions:**

Simulation-based “SNP-environment barcodes” may be used to predict the risk of occurrence of oral malignant disorders. Applying simulation-based “SNP-environment barcodes” may provide insight into the importance of screening tests in preventing oral and pharyngeal cancers and OPMD.

## Introduction

Oral malignant disorders include cancers of the oral cavity and pharynx and oral potentially malignant disorders (OPMD), such as leukoplakia, oral submucous fibrosis (OSF), verrucous hyperplasia, and erythroplakia [1]. In the world, oral and pharyngeal cancers is the sixth most prevalent cancer [2], and patients with OPMD may experience malignant transformation into oral and pharyngeal cancers [3–6].

Environmental carcinogens and genetic polymorphisms (single-nucleotide polymorphisms (SNPs)) are essential components in oral malignant disorder expression. Alcohol consumption, betel quid (BQ) use, and cigarette consumption are significantly related to the risk of developing oral malignant disorders, and a joint effect has been observed when several of these factors are expressed simultaneously [7–10]. Previous studies have demonstrated that environmental and genetic factors are associated in the carcinogenesis of malignant disorders [11, 12]. For example, some oral cancer studies have demonstrated that phase I cytochrome P450 (*CYP*) enzymes may be associated with the metabolic activation of environmental carcinogens [10, 13–16].

In the human genome, SNPs are the most common sequence variations [17]. SNPs are widely used as suitable markers in association studies of several diseases [18], cancers [19], and in pharmacogenomics [20]. Recently, studies have suggested that SNPs of *CYP26* family genes, such as *CYP26A1*, *CYP26B1*, and *CYP26C1*, were associated with susceptibility to oral and pharyngeal cancers [21, 22]. Although genes encoding *CYP* enzymes and environmental factors have been separately evaluated for their effects on the risk of developing oral malignant disorders in previous studies, the combined effect of these genes and environmental factors requires further investigation.

Our previous studies have illustrated that evolutionary algorithms (such as binary particle swarm optimization (BPSO) with an odds ratio (OR)-based method and genetic algorithm) can be used to analyze the associations of multiple SNPs [23–28]. Evolutionary algorithms have been used to combine SNPs with genotypes, namely SNP barcodes, e.g. GG, GT, and TT for a SNP with G/T polymorphism [23–28]. These SNPs are aligned with environmental factors, which provide the maximal risk difference of occurrence between the case and control groups and can forecast the susceptibility of the risks of oral diseases (e.g., oral malignant disorders) [23, 24]. Indeed, we refer to these associations as “SNP-environment barcodes,” which can be used to predict the risks of disease. Our method is based on the concept of simulation barcoding to evaluate the risks of oral malignant disorders using SNP-environment combinations [23–28].

Moreover, BPSO of the OR-based method can decide the best SNP-environment barcodes without computing each combination separately and can present the optimal SNP-environment barcodes, which are regarded as a new quantitative measure with maximal statistical difference between case and control groups [27]. This study aimed to apply a powerful OR-based BPSO method to solve the issue related to the simultaneous analysis of multiple independent SNPs and environmental factors that are associated with oral malignant disorders.

## Material and methods

### Participants and data collection

A case-control study (N = 576) was conducted to analyze the risk of occurrence of oral malignant disorders. This study included oral and pharyngeal cancers (n = 242), OPMD (n = 70), and health controls with high prevalence of BQ chewing (n = 264). We identified polymorphisms of *CYP26* family genes by searching the SNP database and conducted a hospital-based case-control study to verify these SNP variants and environmental factors are associated with oral malignant disorders. Data from male patients diagnosed with oral/pharyngeal cancers were collected from the Kaohsiung Medical University Hospital in Taiwan. Based on an oral health survey, healthy male subjects were recruited to comprise a control group from a community with high prevalence of BQ chewing. Written informed consent were signed for all subjects and voluntarily provided whole blood samples. This study was approved and ascertained by the Institutional Review Board of Kaohsiung Medical University Hospital (KMUH-IRB-950315 and KMUH-IRB-20110031). Ethnic categories were classified as Minnan, Hakka, and Taiwanese aborigines. Education levels were classified as ≤6 and >6 years of education. Data pertaining to substance use status, including alcohol drinking, BQ chewing, and cigarette use, and demographic characteristics were collected by professionally trained interviewers. An alcohol drinker was defined as someone who drank alcoholic beverages (regardless of volume) at least once per week for more than 1 year, BQ chewers were defined as those who chewed at least one quid per day for more than a year, and cigarette smokers were defined as those who smoked at least 10 cigarettes per week for more than 1 year.

### SNP-SNP association problem

An OR is one of the main tools for quantifying the association between exposure and outcome in a given population. Furthermore, OR is most commonly applied in case-control studies to assess the combination of risk factors. In this study, the OR was estimated as follows:

$$OR=\frac{D_{E}\times H_{N}}{H_{E}\times D_{N}}$$ (1)

where *D*_*E*_ is the number of exposed individuals with disease, *H*_*N*_ is number of healthy individuals who were not exposed, *H*_*E*_ is number of healthy individuals who were exposed, and *D*_*N*_ is the number of individuals with a disease who were not exposed. The Pearson chi-square statistic is used to identify factor × factor interaction, which in turn was used to assess significance (*p* < 0.05) in this study. This model measures the interaction between factors (SNPs or environmental factors) and status (genotype or situation) in a two-way contingency table. The estimation value of the test-statistic is shown below:

$$\chi =\sum \frac{{\left(observed-expected\right)}^{2}}{expected}$$ (2)

### Binary particle swarm optimization (BPSO)

BPSO [29, 30] is a swarm intelligence-based optimizer inspired by the social behaviors of birds in finding food. In this method, a particle simulates a bird seeking the best solution in an established space for a particular problem. Assuming that a swarm comprises *N* particles that are moving in a *D*-dimensional search space, the population position and velocity vectors are represented as ***X***∈(***x***_1_, ***x***_2_, … ***x***_*N*_) and ***V***∈(***v***_1_, ***v***_2_, … ***v***_*N*_), respectively. The velocity and position of the *i*th particle are described as ***x***_*i*_ = (*x*_*i*1_, *x*_*i*2_, …, *x*_*iD*_) and ***v***_*i*_ = (*v*_*i*1_, *v*_*i*2_, …, *v*_*iD*_) and are constrained by ***x***_*i*_ ∈ [*x*_min_, *x*_max_]^*D*^ and ***v***_*i*_ ∈ [*v*_min_, v_max_] ^*D*^, respectively. The particle moves according to the individual best solution *pBest*_*i*_ and global best solution *gBest* in each iteration. The particles of BPSO ***X*** and ***V*** were initialized by uniform random values and each particle is a candidate solution for a particular optimization problem. Each particle searches for optimized solutions according to the update formula in the topological space neighborhood in each generation.

Originally, BPSO was developed for the continuous optimization of problems; however, it has not been suitable for discrete optimization of problems. Therefore, the BPSO algorithm, which is an improved form of BPSO, was proposed for solving discrete optimization problems [31]. In the binary version, each particle position ***x***_*i*_ has been redefined as encoding an integer, e.g. ***x***_*i*_ ∈ {0, 1}. The position update formula is described as follows:

$$v_{id}=v_{id}+c_{1}r_{1}({pBest}_{id}-x_{id})+c_{2}r_{2}({gBest}_{d}-x_{id})$$ (3)

$$if\;v_{id}\notin (v_{min},v_{max})\;then\;v_{id}=\max\left(\min\left(v_{max},v_{id}\right),v_{min}\right)$$ (4)

$$S(v_{id})=\frac{1}{1+e^{-v_{id}}}$$ (5)

$$if\;(S(v_{id})>r_{3})\;then\;x_{id}=1;else\;x_{id}=0$$ (6)

where *d* = 1, 2, …, *D*. *c*_1_ and *c*_2_ are the constant for acceleration coefficients. *r*_1_, *r*_2_, and *r*_3_ are the unique random values between [0, 1] at each iteration for each individual dimension. S () is a sigmoid function, which limits the transformation value from 0 to 1.

### BPSO for gene (G×G)/environment (E×E) interaction analysis

This section introduces a BPSO algorithm to address the SNP-SNP interaction problem. We improve the fitness function to identify the interaction between OPMD vs. normal and oral cancer vs. normal. The description is written as follows and includes the encoding schemes, fitness function, and BPSO procedure.

### Encoding schemes

Each particle was designed to be binary-encoding and express a particular number of factor *F* combinations. The particle position is described as *x*_*i*_ = {(*F*_11_, *F*_12_), (*F*_21_, *F*_22_), …, (*F*_*M*1_, *F*_*M*2_)}, where *M* represents the number of factors and (*F*_*m*1_, *F*_*m*2_) shows the selection of the *m*^th^ factor in the *i*^th^ particle in which *F*_*m*1_ and *F*_*m*2_ denote the expression of the particle. These two expressions are based on 0 and 1 variables; thus, four different combinations: (0, 0), (0, 1), (1, 0) and (1, 1) are included. Each particle as a candidate solution denotes the status of whole factors (selected or non-selected) and separates into three different statuses. The four different combinations are shown below:

$$(F_{m1},F_{m2})=\left\{ \begin{array}{l} (0,0),non-selected\\ (0,1),selected\;mth\;factor\;and\;status\;as\;1\\ (1,0),selected\;mth\;facotr\;and\;status\;as\;2\\ (1,1),selected\;mth\;factor\;and\;status\;as\;3 \end{array}\right.$$ (7)

Based on formula (7), four different combinations of (*F*_*m*1_, *F*_*m*2_) can display the selection states of a factor. For instance, a particle in position *x*_*i*_ = {(0, 1), (0, 0), (1, 1), (1, 0), (0, 0)} indicates the selection state to be the 1^st^, 3^rd^, and 4^th^ factor in status 1, 3 and 2, respectively. Consequently, this expression can be used to represent multiple order interactions.

### Fitness function

The best combination of clinical diagnoses for oral cancer and OPMD can be found as a reference to confirm the fitness function of each candidate. We utilized OR, chi-square model, and the OR difference between oral cancer vs. normal and OPMD vs. normal to identify their risk factors. The fitness function formula can be written as:

$$f(x_{i})=S({OR}_{CvN})+S({OR}_{PvN})+S({OR}_{CvN}-{OR}_{PvN})+p$$ (8)

where *S* () is the sigmoid function, which limits [0, 1] each weight and establishes the weight equilibrium, OR_CvN_ represents the OR value in oral cancer and normal cases, and OR_PvN_ represents OR value in OPMD and normal cases. *p* indicates the Pearson chi-squared module to assess the significance in OR_CvN_ and OR_PvN_, which is shown as formula (9).

$$if\;{p‐value}_{CvN}<0.05\;and\;{p‐value}_{PvN}<0.05\;then\;p=1\;else\;p=0$$ (9)

### BPSO procedure

This study utilized BPSO to analyze interaction processes. The detailed BPSO procedure is described as follows.

Step 1) Initialize the population of particles with a uniform random position within {0, 1} and velocity within [*v*_min_, *v*_max_].

Step 2) Calculate the fitness of each particle using the OR value, p-value, and the OR difference according to the Eq (8).

Step 3) Update the individual and global best solution pBest and gBest according to the fitness estimation results.

Step 4) Calculate the particle velocity and update the position of each particle according to Eqs (3)–(6).

Step 5) Repeat Steps 2–4 until the stop criterion has been met. The best combination of clinical diagnosis for risk factor of oral cancer and OPMD is consequently obtained.

### Genotyping of *CYP*26 families

Peripheral blood (8 mL) was drawn from the subjects in ethylenediaminetetraacetic acid (EDTA) tubes. Using standard protocol, genomic DNA was extracted from peripheral blood lymphocytes and immediately stored at –20°C for further analysis. SNPs of *CYP26A1*, *CYP26B1*, and *CYP26C1* with minor allele frequency (MAF) were selected from a public reference Chinese HapMap database, CHB. Therefore, *CYP26A1*, *CYP26B1*, and *CYP26C1* SNPs were selected with MAF > 10% variants from HapMap-CHB. In addition to MAF >10%, our previous study also indicated that the variants of *CYP26A1* rs4411227 were significantly related to an increased risk of malignant oral disorders [21]. As mentioned above, we only selected one rs4411227 for *CYP26A1* SNP analysis in this study.

The assay of TaqMan SNP genotyping (Applied Biosystems, Foster City, CA, USA) was used to test genotypes. DNA samples and negative controls were loaded and examined in 96-well plates using the real-time polymerase chain reaction (PCR) system of ViiA™ 7 Biosystems (Applied Biosystems, Foster City, CA). Using the Sequence Detection System (SDS) 2.1 software (Applied Biosystems) fluorescence data were evaluated, and the fluorescence signals were plotted to determine the genotype of each sample.

### Statistical analysis

We conducted an OR-based BPSO (python algorithm program) and chi-square test to examine the association between the habits of substance use (alcohol drinking, BQ chewing, and cigarette smoking) and disease groups (OPMD, oral/ pharyngeal cancer and normal controls). Chi-square statistical analyses were performed using the SAS statistical package (version 9.1.3, SAS Institute Inc.). The application performance of SNP-environment barcodes was ascertained by receiver operating characteristic (ROC) curve and maximized Youden’s index (sensitivity + specificity– 1) was used to determine optimal cut-off points of OR scores for oral malignant disorders between low and high risk populations [32, 33].

All variables in this study were available on a public repository website (https://github.com/kuochuanwu/SNPbarcode) and my minimal data (filename: 190618 CYP26 SNPs-environment_risk.xlsx) set as the supplementary data, S1 Table.

## Results

### Characterization of the study population

**Fig 1:**
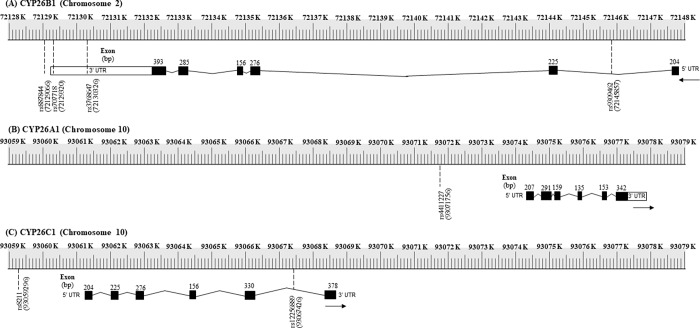
The genetic landscapes of CYP26 SNPs. (A) Four candidate SNPs are indicated in genomic structure of *CYP26B1* (2p13.2). SNP rs887844 is located in 500B downstream variant, followed by rs707718 in 3’ UTR, rs3768647 in 3’ UTR, and rs9309462 in intron. (B) One candidate SNP is indicated in genomic structure of *CYP26A1* (10q23.33). SNP rs4411227 is located in 2KB upstream variant. (C) Two candidate SNPs are indicated in genomic structure of *CYP26C1* (10q23.33). SNP rs8211 is located in 2KB upstream and SNP rs12256889 is located in intron. Exons are denoted by black boxes.

The gene structure and SNP information of *CYP26* gene families are shown in Tables 1 and 2. and genetic landscape of these SNPs was described in Fig 1. There were no non-synonymous variants (amino acid changes) from these SNPs. In the aspect of *CYP26B1* (2p13.2), four candidate SNPs are indicated. SNP rs887844 is located in 500B downstream variant, followed by rs707718 in 3’ UTR, rs3768647 in 3’ UTR, and rs9309462 in intron. We selected only one SNP rs4411227 for *CYP26A1* on chromosome 10q23.33 region and rs4411227 is located in 2KB upstream variant. In the region of *CYP26C1* (10q23.33), SNP rs8211 is located in 2KB upstream and SNP rs12256889 is located in intron.

**Table 1:** CYP26 gene structure for association study of oral malignant disorders.

Chromosome	Gene	Region	Chromosome Position^a^		Region Length
			start	end	
2p13.2	CYP26B1				
		UTR	72,147,835	72,147,862	
		Exon	72,147,631	72,147,834	204
		Exon	72,143,989	72,144,213	225
		Exon	72,135,144	72,135,419	276
		Exon	72,134,761	72,134,916	156
		Exon	72,133,023	72,133,307	285
		Exon	72,132,227	72,132,619	393
		UTR	72,129,238	72,132,226	
10q23.33	CYP26A1				
		UTR	93,074,307	93,074,325	
		Exon	93,074,326	93,074,532	207
		Exon	93,074,779	93,075,069	291
		Exon	93,075,149	93,075,307	159
		Exon	93,075,826	93,075,960	135
		Exon	93,076,544	93,076,696	153
		Exon	93,076,963	93,077,304	342
		UTR	93,077,305	93,077,884	
10q23.33	CYP26C1				
		UTR	93,061,263	93,061,263	
		Exon	93,061,264	93,061,467	204
		Exon	93,062,010	93,062,234	225
		Exon	93,062,720	93,062,995	276
		Exon	93,064,381	93,064,536	156
		Exon	93,065,956	93,066,285	330
		Exon	93,068,320	93,068,697	378
		UTR	93,068,698	93,068,697	

^a^ The data was cited from website http://www.rcsb.org/pdb/gene/ (GRCh38)

**Table 2:** CYP26 SNP information for association study of oral malignant disorders.

SNP No.	Chromosome	Chromosome Position^a^	Gene	SNP rs No.	Location^a^	Genotype		
						0	1	2
1	2	72129066	CYP26B1	rs887844	500B Downstream Variant	-	A/G	G/G
2	2	72129320	CYP26B1	rs707718	3’ UTR	G/G	G/T	T/T
3	2	72130326	CYP26B1	rs3768647	3’ UTR	-	C/G	G/G
4	2	72145857	CYP26B1	rs9309462	Intron Variant	-	C/T	T/T
5	10	93071756	CYP26A1	rs4411227	2KB Upstream Variant	C/C	C/G	G/G
6	10	93059296	CYP26C1	rs8211	2KB Upstream Variant	C/C	C/T	T/T
7	10	93067426	CYP26C1	rs12256889	Intron Variant	-	A/C	C/C

^a^ The data was cited from GRCh38.p12, National Center for Biotechnology Information (NCBI) (https://www.ncbi.nlm.nih.gov/pubmed/).

3’ UTR, three prime untranslated region.

The demographic characteristics of and *CYP26* polymorphism in individuals with oral/pharyngeal cancers (N = 242), OPMD (N = 70) and in control subjects (N = 264) with high prevalence of BQ chewing are described in Table 3. Five hundred and seventy-six individuals participated in our study. All male participants were over 18 years of age (mean age 48.29 ± 9.88 years). The mean age of control individuals was 44.49 ± 8.53 years, 51.01 ± 11.82 years for patients with OPMD, and 51.64 ± 9.17 years for patients with oral and pharyngeal cancer. Results demonstrated statistically significant age differences among the three groups. In terms of age, the oral and pharyngeal cancer group contained more patients (52.89%) aged over 50, which was higher than that in the controls (21.97%) significantly. Moreover, 55.71% patients with OPMD were aged over 50, which was higher than the controls group (21.97%). The percentage of individuals with low education level was 34.30% in patients with oral and pharyngeal cancers, which was significantly higher than that of the controls (22.35%). The prevalence of consuming alcohol, chewing BQ, and smoking cigarettes was not statistically significant among patients with oral/pharyngeal cancer and OPMD and the control group. According to the results of genotyping analysis, there was no deviation for all seven SNPs from the Hardy-Weinberg equilibrium in both the oral malignant disorder and control groups. The results showed that genotype frequency for *CYP26A1* (rs4411227), and *CYP26B1* (rs887844, rs3768647, and rs9309462) were significantly different among the three groups (*p* < 0.05).

**Table 3:** Distribution of selected demographic characteristics and the genotypes of CYP26 families among oral and pharyngeal cancers, OPMD, and controls (N = 576).

Variables	Oral and pharyngeal cancers (N = 242)		OPMD (N = 70)		Controls (N = 264)		*p*-value^a^
	N	(%)^b^	N	(%)	N	(%)	
**Environment**							
Sex							
1 male	232	(95.87)	69	(98.57)	264	(100.00)	0.003
2 female	10	(4.13)	1	(1.43)	0	(0.00)	
Age, year (mean ± SD)	51.64 ± 9.17		51.01 ± 11.82		44.49 ± 8.53		<0.001^c^
1 ≤ 50	114	(47.11)	31	(44.29)	206	(78.03)	<0.001
2 > 50	128	(52.89)	39	(55.71)	58	(21.97)	
Race							
1 Minnan	202	(83.47)	53	(75.71)	209	(79.17)	0.261
2 Other	40	(16.53)	17	(24.29)	55	(20.83)	
Education level							
1 ≤ 6 year	83	(34.30)	21	(30.00)	59	(22.35)	0.011
2 > 6 year	159	(65.70)	49	(70.00)	205	(77.65)	
Alcohol drinking							
0 No	86	(35.54)	25	(35.71)	76	(28.79)	0.222
1 Yes	156	(64.46)	45	(64.29)	188	(71.21)	
Betel quid chewing							
0 No	32	(13.22)	9	(12.86)	44	(16.67)	0.492
1 Yes	210	(86.78)	61	(87.14)	220	(83.33)	
Cigarette smoking							
0 No	28	(11.57)	9	(12.86)	19	(7.20)	0.162
1 Yes	214	(88.43)	61	(87.14)	245	(92.80)	
**CYP26 SNPs**^d^							
CYP26A1(rs4411227)							
0 C/C	8	(3.31)	3	(4.29)	6	(2.27)	0.010
1 C/G	81	(33.47)	19	(27.14)	53	(20.08)	
2 G/G	153	(63.22)	48	(68.57)	205	(77.65)	
CYP26B1 (rs887844)							
1 A/G	115	(47.52)	39	(55.71)	99	(37.50)	0.008
2 G/G	127	(52.48)	31	(44.29)	165	(62.50)	
CYP26B1 (rs707718)							
0 G/G	45	(18.60)	10	(14.29)	28	(10.61)	0.161
1 G/T	117	(48.35)	36	(51.43)	139	(52.65)	
2 T/T	80	(33.06)	24	(34.29)	97	(36.74)	
CYP26B1 (rs3768647)							
1 C/G	122	(50.41)	42	(60.00)	264	(100.00)	<0.001
2 G/G	120	(49.59)	28	(40.00)	0	(0.00)	
CYP26B1 (rs9309462)							
1 C/T	17	(7.02)	4	(5.71)	3	(1.14)	0.003
2 T/T	225	(92.98)	66	(94.29)	261	(98.86)	
CYP26C1 (rs8211)							
0 C/C	159	(65.70)	44	(62.86)	198	(75.00)	0.133
1 C/T	74	(30.58)	23	(32.86)	60	(22.73)	
2 T/T	9	(3.72)	3	(4.29)	6	(2.27)	
CYP26C1 (rs12256889)							
1 A/C	234	(96.69)	67	(95.71)	258	(97.73)	0.617
2 C/C	8	(3.31)	3	(4.29)	6	(2.27)	

^a^ Significant difference was test by Chi-square analysis (*p* < 0.05).

^b^ May not total 100% due to rounding.

^c^ Significant difference was test by general linear (GLM) model (*p* < 0.05). SD, standard deviation.

^d^ The genotype information of case and control was partly derived from our previous work [21, 22] and variables were available at https://github.com/kuochuanwu/SNPbarcode (S1 Table).

### Association between combined polymorphisms of *CYP26* and oral malignant disorders

After considering age, gender, race, education, alcohol consumption, BQ chewing, and cigarette smoking, the joint effect (adjusted OR and 95% CI) of specific SNP combinations and environmental factors on the occurrence of oral malignant disorders were obtained (Table 4). Specific SNP (*CYP26B1* rs887844 (A/G), *CYP26A1* rs4411227 (C/G), *CYP26C1* rs12256889 (A/C), and *CYP26B1* rs707718 (G/T)) and environmental factor (> 50 years old) combinations significantly increased the risks (5.75-, 3.92-, 4.07-, and 3.34-fold, respectively) for patients with OPMD compared to health controls with high prevalence of BQ chewing. Patients with oral and pharyngeal cancers and specific SNP (*CYP26B1* rs887844 (A/G), *CYP26A1* rs4411227 (C/G), *CYP26C1* rs12256889 (A/C), and *CYP26B1* rs707718 (G/T)) and environmental factor (> 50 old) combinations exhibited significantly increased risks (6.76-, 4.16-, 3.94-, and 2.80-fold, respectively) compared to health controls with high prevalence of BQ chewing. Elder subjects with rs887844 (A/G) or rs4411227 SNP (C/G) were more at risk of developing oral and pharyngeal cancers than OPMD. Inversely, elder subjects with rs12256889 (A/C), rs4411227 SNP (G/G) or rs707718 (G/T) SNPs were at lower risk of developing oral and pharyngeal cancers than OPMD. Thus, our results suggest the presence of a genome-wide cross-talk between polymorphisms of several genes and SNPs of *CYP26*, which may be involved in the occurrence of oral malignant disorders.

**Table 4:** The top ten best risk association models of 2 factors combinations among oral and pharyngeal cancers, OPMD, and controls.

		OPMD vs controls					Oral and pharyngeal cancers vs controls					
Model	Combination	Cases	Controls	OR	*p*-value	95% CI	Cases	Controls	OR	*p*-value	95% CI	△^a^
Age, rs887844	Other	52	249				172	249				
	2–1	18	46	5.75	<0.001	2.72–12.13	70	15	6.76	<0.001	3.74–12.19	1.01
Age, Race	Other	40	218				132	218				
	2–1	30	46	3.55	<0.001	2.01–6.29	110	46	3.95	<0.001	2.63–5.93	0.40
Age, rs4411227	Other	59	252				202	252				
	2–1	11	12	3.92	0.003	1.65–9.31	40	12	4.16	<0.001	2.13–8.14	0.24
Age, Alcohol drinking	Other	54	242				186	242				
	2–0	16	22	3.26	0.001	1.61–6.62	56	22	3.31	<0.001	1.95–5.62	0.05
Age, rs12256889	Other	33	207				116	207				
	2–1	37	57	4.07	<0.001	2.34–7.08	126	57	3.94	<0.001	2.68–5.81	-0.13
Age, rs4411227	Other	44	220				157	220				
	2–2	26	44	2.95	<0.001	1.65–5.29	85	44	2.71	<0.001	1.78–4.11	-0.24
Age, Betel quid chewing	Other	39	221				141	221				
	2–1	31	43	4.09	<0.001	2.30–7.25	101	43	3.68	<0.001	2.43–5.57	-0.41
Age, Alcohol drinking	Other	47	228				170	228				
	2–1	23	36	3.10	<0.001	1.68–5.71	72	36	2.68	<0.001	1.72–4.19	-0.42
Age, rs707718	Other	49	234				178	234				
	2–1	21	30	3.34	<0.001	1.77–6.32	64	30	2.80	<0.001	1.74–4.51	-0.54
Age, Cigarette smoking	Other	37	216				138	216				
	2–1	33	48	4.01	<0.001	2.28–7.05	104	48	3.39	<0.001	2.27–5.08	-0.62

^a^ The difference between oral and pharynx cancers vs control and OPMD vs control.

OR, odds ratio. 95% CI, 95% confidence interval.

### Best combination model of SNP-environment with maximum risk differences between cases and controls

We used the BPSO-generated barcodes to compute the relative strength of SNP-environment combination effects on the risk of developing oral malignant disorders and determined the risk effects of subjects with oral malignant disorders who exhibited specific SNP-environment combinations or other combinations (Table 5). After using controls (high prevalence of BQ chewing) as a reference group, we found that the elderly people within the Minnan population with SNP rs887844 (A/G) were more likely to develop oral and pharyngeal cancers (OR = 8.09) compared with OPMD (OR = 5.75). Elderly Minnan individuals who chewed BQ and harbored the SNP rs887844 (A/G) showed an elevated risk of oral and pharyngeal cancers (OR = 10.3) compared with OPMD (OR = 6.12). The maximum risks difference (oral and pharyngeal cancers versus the control groups (OR = 10.3) − OPMD versus the control group (OR = 5.42)) of 4.88 was found in elderly Minnan individuals who chewed BQ and carried the SNPs rs887844 (A/G) and rs12256889 (A/C).

**Table 5:** Estimated joint effects on models of environmental-SNP combinations associated with oral and pharyngeal cancers, OPMD, and controls.

		OPMD vs controls							Oral and pharyngeal cancers vs controls							
Model	Combinations	Cases (%)		Controls (%)		OR	*p*-value	95% CI	Cases (%)		Controls(%)		OR	*p*-value	95% CI	△^a^
Age, rs887844	Other	52	(74.29)	249	(95.77)				172	(71.07)	249	(94.32)				
	2–1	18	(25.71)	11	(4.23)	5.75*	0.000	2.72–12.13	70	(28.93)	15	(5.68)	6.76*	0.000	3.74–12.19	1.01
Age, Race, rs887844	Other	56	(80.00)	253	(95.83)				179	(73.97)	253	(95.83)				
	2-1-1	14	(20.00)	11	(4.17)	5.75*	0.000	2.48–13.33	63	(26.03)	11	(4.17)	8.09*	0.000	4.15–15.80	2.34
Age, Race, BQ chewing, rs887844	Other	60	(85.71)	257	(97.35)				189	(78.10)	257	(97.35)				
	2-1-1-1	10	(14.29)	7	(2.65)	6.12*	0.000	2.24–16.73	53	(21.90)	7	(2.65)	10.3*	0.000	4.58–23.15	4.18
Age, Race, BQ chewing, rs887844, rs12256889	Other	61	(87.14)	257	(97.35)				189	(78.10)	257	(97.35)				
	2-1-1-1-1	9	(12.86)	7	(2.65)	5.42*	0.001	1.94–15.12	53	(21.90)	7	(2.65)	10.3*	0.000	4.58–23.15	4.88
Age, Race, BQ chewing, Cigarette smoking, rs887844, rs12256889	Other	62	(88.57)	257	(97.35)				193	(79.75)	257	(97.35)				
	2-1-1-1-1-1	8	(11.43)	7	(2.65)	4.74*	0.005	1.66–13.56	49	(20.25)	7	(2.65)	9.32*	0.000	4.13–21.03	4.58

^a^ The risk difference between oral and pharynx cancers vs controls and OPMD vs controls.

OR, odds ratio. 95% CI, 95% confidence interval.

* An OR value ≥ 2.39 indicates high risk with oral malignant disorders.

**Fig 2:**
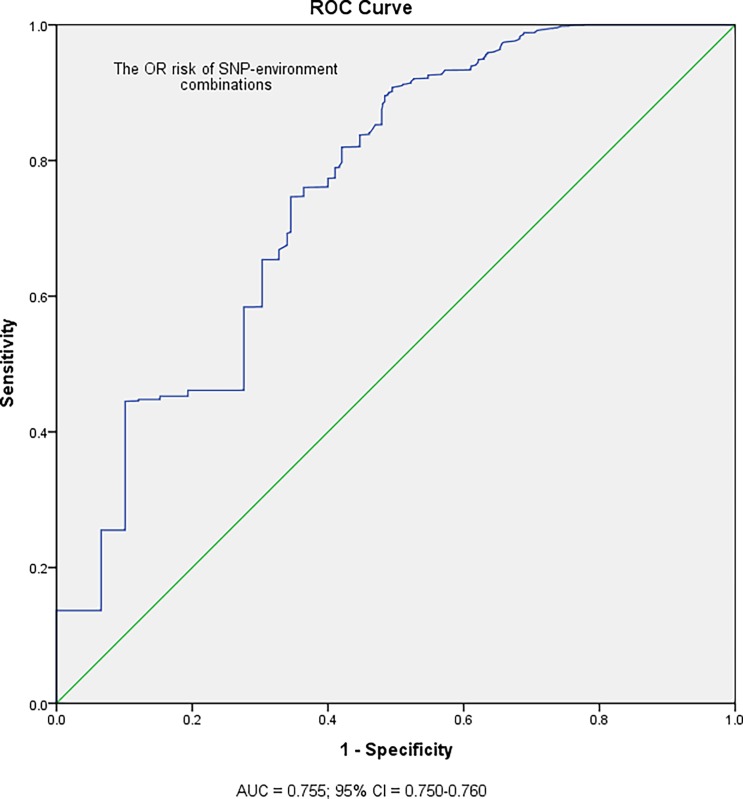
Receiver operating characteristic (ROC) curve analysis of the OR risks of SNP-environment combinations for population with oral malignant disorders. The area under the curve (AUC) = 0.755 (95% confidence interval (CI) = 0.750–0.760; *p*<0.001) was used to discriminate OR *<* 2.39 from OR ≥ 2.39 for low and high risk populations with oral malignant disorders.

In the risks assessment, the odds ratio (OR) was used to evaluate the impact of risk association of SNP-environment combinations for oral malignant disorders between high and low risk populations. We used ROC curve to predict the discriminatory power of OR risks of SNP-environment barcodes for population with oral malignant disorders (Fig 2). A significant application performance (area under the curve (AUC) = 0.755; 95% confidence interval (CI) = 0.750–0.760; *p*<0.001) of SNP-environment barcodes for oral malignant disorders was found by ROC curve. Maximized Youden’s index was used to determine optimal cut-off points of OR scores for predicting high risk population with oral malignant disorders. Optimal sensitivity (91%) for predicting high risk population was achieved with a OR cut-off of greater than or equal to 2.39. Indeed, we provided the best discrimination of OR < 2.39 and OR ≥ 2.39 for oral malignant disorders between low and high risk populations.

## Discussion

The occurrence of cancers was implicated in the combined effects between genes and environment, but confirming joint effects was a computational and mathematical challenge. In terms of multiple SNPs, genome-wide association studies (GWAS) have been indicated commonly to discover the associative effects of diseases [34–39]. A number of GWAS [40–42] and non-GWAS [43, 44] studies have recognized the interaction of SNPs. Nevertheless, the association effects based on SNP-SNP interaction between *CYP* families SNPs and the risk associations in oral malignant disorders are less frequently mentioned. In terms of the computational biological challenge, potential and substantial associations are often hidden in the large number of possible combined effects on numerous SNPs genotypes. A number of studies have indicated computational approaches to ameliorate the effects of association of multiple SNPs analysis [45–49]. Nevertheless, these strategies have been confronted with computational intensity as the number of SNPs increases [50]. Major issues arise from this challenge, along with calculating SNP-SNP relationships in terms of combinations of SNPs corresponding to genotypes and SNPs combined with environmental factors.

The approaches of conventional statistics, machine learning, and data mining have been established to evaluate potential effects in GWAS association studies [24, 25, 51–56]. Therefore, mathematical optimization algorithm of the OR-based method was applied to examine the susceptible risks of several cancers and disorders [23, 27, 28, 53, 55, 57–59]. The strength of BPSO was used to ascertain the simulation-based association models with rapid and easy statistically analysis.

Epidemiological studies indicated that OPMD and oral/pharyngeal cancers have a close relationship with environmental exposure to BQ, alcohol, and cigarettes, particularly in heavy BQ users [7, 8, 22, 60–62]. In 2004, IARC indicated that the areca nut and chewable BQ, particularly those without tobacco, comprised group 1 human carcinogens of the oral cavity [9]. In Taiwan, the 2016 cancer report indicated that oral cavity and pharyngeal cancers were the fourth most prevalent cancers with an incidence rate of 42.43 per 100,000 individuals among males [63]. In addition, it ranked as the fourth leading cause of death due to cancer, and the mortality rate was 15.71 per 100,000 individuals [63].

BQ alkaloids, arecoline and arecaidine can cause bacterial mutagenicity, and in mammalian cells, *in vivo* or *in vitro* tests can result in sister chromatographic exchange, chromosomal aberrations, and micronuclei formation [9]. In addition to the areca nut extract, arecoline also induces the dysregulation of oral epithelial cells, leading to cell cycle arrest [64]. We previously reported that arecoline (a major alkaloid in BQ) significantly induced *CYP26B1* expression [65]. *CYP26* has three isoforms—namely, *CYP26A1*, *CYP26B1*, and *CYP26C1*—which mainly metabolize retinoic acid (RA)-related compounds [66]. RA is a biologically active derivative of vitamin A, which regulates growth, differentiation, and apoptosis of several cell types, and plays an important role in visual physiological function, embryonic development patterns, and adult physiological mechanisms [67, 68].

The risk of developing oral malignant disorders may be related to the functionally relevant combined effects of SNPs such as those in *CYP26A1*, *CYP26B1*, and *CYP26C1* within and between different cancer pathways. Since interactions among multiple genes influenced the risk component of oral and pharyngeal cancers, our rationale for identifying interactions between genes was justified. We developed new and feasible analytical methods to systematically examine the interactions between genome-wide SNPs and various environmental factors. In particular, we investigated the role of combinational SNPs in three metabolism-related genes (*CYP26A1*, *CYP26B1*, and *CYP26C1*) in oral malignant disorders. In association studies of disease predisposition, the interaction analyses of SNPs increased the performance [19, 28, 58, 69–71].

In oral and pharyngeal cancers and OPMD disorders, we also explored the risk factors for genetic variation of complex traits. We hypothesized that two important SNPs (*CYP26B1* rs887844 and *CYP26C1* rs12256889) within *CYP26* may significantly elevate genetic susceptibility to oral and pharyngeal cancers and OPMD. The association between the risk of developing oral/pharyngeal cancers and OPMD and *CYP26* SNPs was detected by a robust BPSO algorithm combined with statistical analysis. The BPSO algorithm optimally evaluated the risk effects of *CYP26* SNPs for oral and pharyngeal cancers and OPMD. Complex multifactor associations are difficult to analyze statistically [72]. Accordingly, comprehensive approaches to evaluate the best association models with disorder-related factors were used in several studies [54, 56, 73, 74], and these methods have suitable power to test the potential model of associations.

SNPs and environmental factor combinations were calculated using the BPSO method, and their relationships with disease risk were examined by selecting prominent SNPs. Indeed, this algorithm assisted in comprehensively recognizing the genetic basis of complex diseases/traits. Our published study presented that *CYP26* is a candidate gene family for assessing the risks of occurrence of oral/pharyngeal cancers and OPMD [10, 21, 22], and specific SNP combinations of *CYP26* may be associated with increased risk of developing oral/pharyngeal cancers and OPMD. Nevertheless, the joint effects of *CYP26* SNP-environmental factor combinations on the risk of developing oral malignant disorders were not examined previously using SNP-environmental factors interaction methods.

In this study, we revealed a strong correlation between *CYP26* SNP-environmental factor interaction (old age, Minnan ethnicity, BQ chewers, and smoking) and the risk of oral malignant disorder occurrence. Two significant *CYP26* SNPs (*CYP26B1* rs887844 (A/G) and *CYP26C1* rs12256889 (A/C)) were selected using BPSO analysis, which was the best performance model for oral malignant disorder occurrence risk analysis. The analysis suggested that several combinations of environmental factors and candidate SNPs had the highest risk for susceptibility to oral malignant disorders. The risks were more prominent in the oral and pharyngeal cancers group (OR = 10.30; 95% CI = 4.58–23.15) than in the OPMD group (OR = 5.42; 95% CI = 1.94–15.12). This finding implies that older individuals with SNP rs887844 and rs12256889 of Minnan ethnicity who chewed BQ were more likely to suffer from oral and pharyngeal cancers than from OPMD.

**Fig 3:**
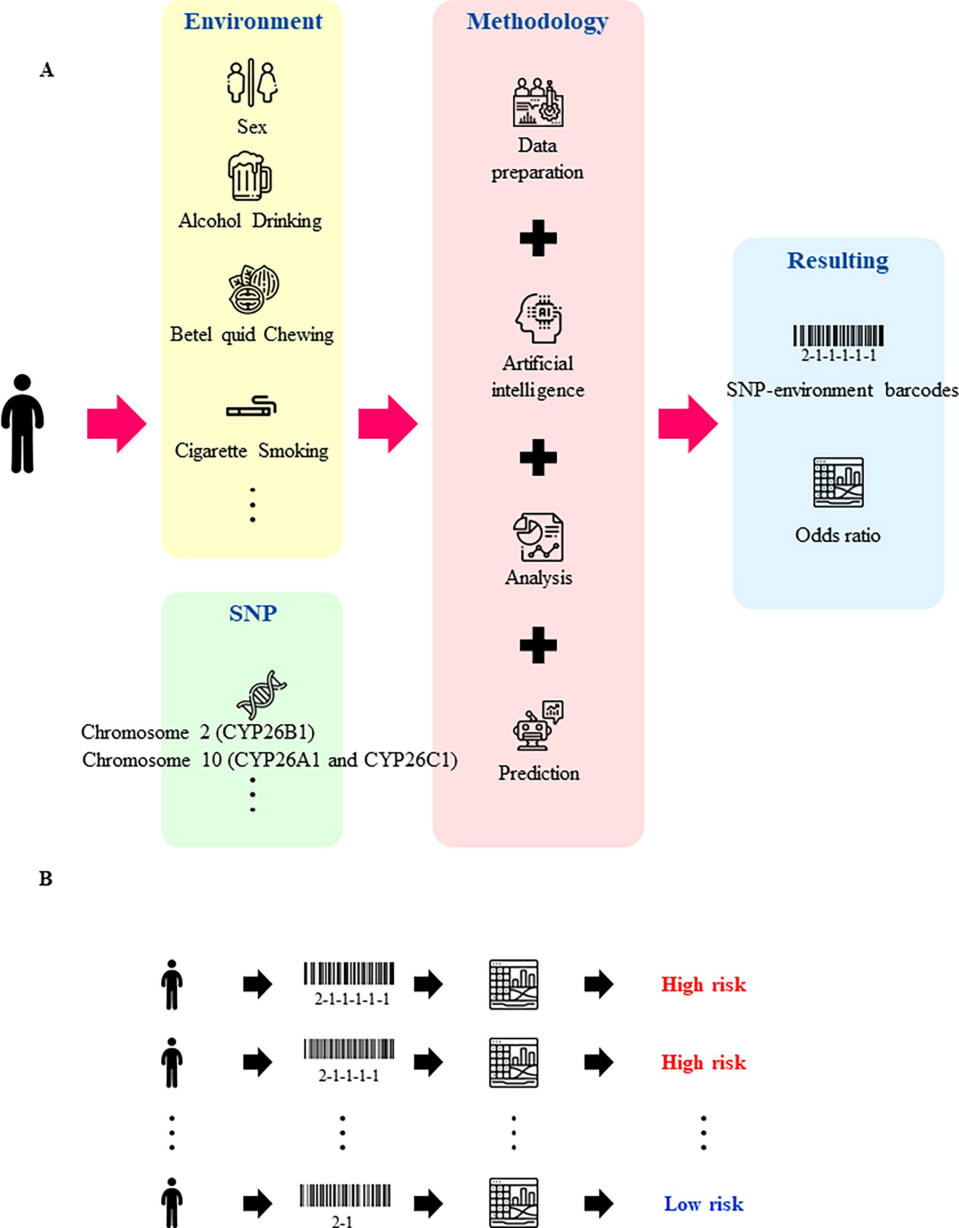
Application of simulation-based novel SNP-environment barcodes for evaluating the occurrence of oral malignant disorders by odds ratio-based BPSO. (A) After calculating and analysis statistically by BPSO, we render combined associations of each high-risk group from SNP-environment data. (B) SNP-environment data can be converted to create barcodes (e.g., 2–1, 2-1-1-1-1, and 2-1-1-1-1-1) from each subject and render them prone to the low and high risk group of oral malignant disorders. ICONs are designed by Freepik (https://www.freepik.com/).

In this study, we conducted a powerful binary particle swarm optimization (BPSO) of an odds ratio (OR)-based method to evaluate the joint effect of gene-gene-environment (SNP-SNP-environment) in oral malignant disorders. The BPSO-based SNP-SNP and SNP-environment interactions were not limited to SNPs on the same chromosome, and our algorithm could control potential confounders and diverse numbers of SNP. Moreover, the BPSO approach calculated the best performance of the SNP or SNP-environment model with the risk of maximum difference between cases and control groups. The ROC curve was used to discriminate OR risks of SNP-environment barcodes for oral malignant disorders as shown in Fig 2. Certainly, the best cutoff points of OR < 2.39 and OR ≥ 2.39 were provided for oral malignant disorders between low and high risk populations. The idea of simulation-based CYP26 SNP-environment barcodes and barcode concepts for population with oral malignant disorders were presented in schematic diagram (Fig 3). Our SNP-environment data can be converted to create barcodes to distinguish between the low and high risk group (e.g., 2–1, and 2-1-1-1-1-1) of oral malignant disorders.

### Study limitations

Although we are do not create a barcode system for evaluating the risks of occurrence of oral malignant disorders, the term “barcodes” has been applied in a number of our published papers [23–28]. Owing to the difficulty of the statistical analyses in evaluating complex multifactor associations (particularly gene jointed with environment factors), we render the OR-based BPSO method to generate SNP-environment barcodes as a simulation proxy of barcodes to predict the risks of oral malignant diseases susceptibility.

After calculations and a statistical analysis using BPSO approach, and we render combined associations of each high-risk group from SNP-environment data (Table 5). We clarified the proposed concept of SNP-environment barcodes as shown in Fig 3. SNP-environment data can be converted to create SNP-environment barcodes from each subject and render them prone to the high risk group (OR ≥ 2.39) of oral malignant disorders.

## Conclusions

We demonstrated the significant joint effects of *CYP26* SNPs and environmental factors on the risk of oral malignant disorder occurrence by BPSO method for the first time. Therefore, the BPSO-based SNP-SNP and SNP-environment approaches for assessing the combined effects of the novel SNP-environment approach may potentially assist in identifying complex biological relationships among cancer processes during the development of oral malignant disorders.

In summary, the combined effects of the novel *CYP26* SNP-environment approach may predict the risk of occurrence of oral malignant disorders. Its application as a proxy of SNP-environment barcodes will provide insights into the importance of establishing screening tests for BQ chewers to promote the prevention of oral malignant disorders.

## Supporting information

S1 Table Supplementary data (190618 CYP26 SNPs-environment_risk). (XLSX)

## References

[1] Warnakulasuriya S, Johnson NW, van der Waal I. Nomenclature and classification of potentially malignant disorders of the oral mucosa. J Oral Pathol Med. 2007;36(10):575–80. Epub 2007/10/20. 10.1111/j.1600-0714.2007.00582.x . 17944749

[2] Warnakulasuriya S. Global epidemiology of oral and oropharyngeal cancer. Oral Oncol. 2009;45(4–5):309–16. 10.1016/j.oraloncology.2008.06.002 . 18804401

[3] Shiu MN, Chen TH, Chang SH, Hahn LJ. Risk factors for leukoplakia and malignant transformation to oral carcinoma: a leukoplakia cohort in Taiwan. Br J Cancer. 2000;82(11):1871–4. 10.1054/bjoc.2000.1208 10839305 PMC2363234

[4] Hsue SS, Wang WC, Chen CH, Lin CC, Chen YK, Lin LM. Malignant transformation in 1458 patients with potentially malignant oral mucosal disorders: a follow-up study based in a Taiwanese hospital. J Oral Pathol Med. 2007;36(1):25–9. 10.1111/j.1600-0714.2006.00491.x . 17181738

[5] Ho PS, Chen PL, Warnakulasuriya S, Shieh TY, Chen YK, Huang IY. Malignant transformation of oral potentially malignant disorders in males: a retrospective cohort study. BMC Cancer. 2009;9:260 10.1186/1471-2407-9-260 19640311 PMC2734864

[6] Wang YY, Tail YH, Wang WC, Chen CY, Kao YH, Chen YK, et al Malignant transformation in 5071 southern Taiwanese patients with potentially malignant oral mucosal disorders. BMC Oral Health. 2014;14:99 10.1186/1472-6831-14-99 25096230 PMC4134123

[7] Ko YC, Huang YL, Lee CH, Chen MJ, Lin LM, Tsai CC. Betel quid chewing, cigarette smoking and alcohol consumption related to oral cancer in Taiwan. J Oral Pathol Med. 1995;24(10):450–3. . 8600280 10.1111/j.1600-0714.1995.tb01132.x

[8] Lee CH, Ko YC, Huang HL, Chao YY, Tsai CC, Shieh TY, et al The precancer risk of betel quid chewing, tobacco use and alcohol consumption in oral leukoplakia and oral submucous fibrosis in southern Taiwan. Br J Cancer. 2003;88(3):366–72. 10.1038/sj.bjc.6600727 . 12569378 PMC2747536

[9] IARC. Betel-quid and areca-nut chewing and some areca-nut-derived nitrosamines: IARC Monogr Eval Carcinog Risks Hum; 2004. PMC4781453 15635762

[10] Chen PH, Lee KW, Hsu CC, Chen JY, Wang YH, Chen KK, et al Expression of a splice variant of CYP26B1 in betel quid-related oral cancer. ScientificWorldJournal. 2014;2014:810561 10.1155/2014/810561 25114974 PMC4119653

[11] Yang CH, Lin YD, Yen CY, Chuang LY, Chang HW. A systematic gene-gene and gene-environment interaction analysis of DNA repair genes XRCC1, XRCC2, XRCC3, XRCC4, and oral cancer risk. OMICS. 2015;19(4):238–47. 10.1089/omi.2014.0121 . 25831063

[12] Dasgupta S, Reddy BM. The role of epistasis in the etiology of Polycystic Ovary Syndrome among Indian women: SNP-SNP and SNP-environment interactions. Ann Hum Genet. 2013;77(4):288–98. 10.1111/ahg.12020 . 23550965

[13] Hung HC, Chuang J, Chien YC, Chern HD, Chiang CP, Kuo YS, et al Genetic polymorphisms of CYP2E1, GSTM1, and GSTT1; environmental factors and risk of oral cancer. Cancer Epidemiol Biomarkers Prev. 1997;6(11):901–5. Epub 1997/11/21. . 9367063

[14] Kietthubthew S, Sriplung H, Au WW. Genetic and environmental interactions on oral cancer in Southern Thailand. Environ Mol Mutagen. 2001;37(2):111–6. Epub 2001/03/14. . 11246217 10.1002/em.1018

[15] Kao SY, Wu CH, Lin SC, Yap SK, Chang CS, Wong YK, et al Genetic polymorphism of cytochrome P4501A1 and susceptibility to oral squamous cell carcinoma and oral precancer lesions associated with smoking/betel use. J Oral Pathol Med. 2002;31(9):505–11. Epub 2002/09/25. . 12269988 10.1034/j.1600-0714.2002.00158.x

[16] Topcu Z, Chiba I, Fujieda M, Shibata T, Ariyoshi N, Yamazaki H, et al CYP2A6 gene deletion reduces oral cancer risk in betel quid chewers in Sri Lanka. Carcinogenesis. 2002;23(4):595–8. Epub 2002/04/19. 10.1093/carcin/23.4.595 . 11960911

[17] Brookes AJ, 1999. The essence of SNPs. The essence of SNPs. Gene 234, 177–186. 1999 10.1016/s0378-1119(99)00219-x 10395891

[18] Yang CH, Lin YD, Chuang LY, Chang HW. Analysis of high-order SNP barcodes in mitochondrial D-loop for chronic dialysis susceptibility. J Biomed Inform. 2016;63:112–9. 10.1016/j.jbi.2016.08.009 . 27507088

[19] Yen CY, Liu SY, Chen CH, Tseng HF, Chuang LY, Yang CH, et al Combinational polymorphisms of four DNA repair genes XRCC1, XRCC2, XRCC3, and XRCC4 and their association with oral cancer in Taiwan. J Oral Pathol Med. 2008;37(5):271–7. 10.1111/j.1600-0714.2007.00608.x . 18410587

[20] Chang HW, Chuang LY, Tsai MT, Yang CH. The importance of integrating SNP and cheminformatics resources to pharmacogenomics. Curr Drug Metab. 2012;13(7):991–9. Epub 2012/05/18. . 22591347 10.2174/138920012802138679

[21] Wu SJ, Chen YJ, Shieh TY, Chen CM, Wang YY, Lee KT, et al Association study between novel CYP26 polymorphisms and the risk of betel quid-related malignant oral disorders. ScientificWorldJournal. 2015;2015:160185 10.1155/2015/160185 25839051 PMC4369936

[22] Chen PH, Lee KW, Chen CH, Shieh TY, Ho PS, Wang SJ, et al CYP26B1 is a novel candidate gene for betel quid-related oral squamous cell carcinoma. Oral Oncol. 2011;47(7):594–600. Epub 2011/06/07. 10.1016/j.oraloncology.2011.04.024 . 21641851

[23] Chang HW, Chuang LY, Ho CH, Chang PL, Yang CH. Odds ratio-based genetic algorithms for generating SNP barcodes of genotypes to predict disease susceptibility. OMICS. 2008;12(1):71–81. Epub 2008/02/13. 10.1089/omi.2007.0036 . 18266556

[24] Yang CH, Chuang LY, Cheng YH, Lin YD, Wang CL, Wen CH, et al Single nucleotide polymorphism barcoding to evaluate oral cancer risk using odds ratio-based genetic algorithms. The Kaohsiung journal of medical sciences. 2012;28(7):362–8. Epub 2012/06/26. 10.1016/j.kjms.2012.02.002 . 22726897 PMC11916800

[25] Yang CH, Lin YD, Chuang LY, Chang HW. Evaluation of breast cancer susceptibility using improved genetic algorithms to generate genotype SNP barcodes. IEEE/ACM Trans Comput Biol Bioinform. 2013;10(2):361–71. Epub 2013/08/10. 10.1109/TCBB.2013.27 . 23929860

[26] Chuang LY, Lane HY, Lin YD, Lin MT, Yang CH, Chang HW. Identification of SNP barcode biomarkers for genes associated with facial emotion perception using particle swarm optimization algorithm. Ann Gen Psychiatry. 2014;13:15 Epub 2014/06/24. 10.1186/1744-859X-13-15 24955105 PMC4050220

[27] Chang HW, Yang CH, Ho CH, Wen CH, Chuang LY. Generating SNP barcode to evaluate SNP-SNP interaction of disease by particle swarm optimization. Comput Biol Chem. 2009;33(1):114–9. Epub 2008/09/16. 10.1016/j.compbiolchem.2008.07.029 . 18789770

[28] Yang CH, Chang HW, Cheng YH, Chuang LY. Novel generating protective single nucleotide polymorphism barcode for breast cancer using particle swarm optimization. Cancer Epidemiol. 2009;33(2):147–54. 10.1016/j.canep.2009.07.001 . 19679063

[29] Kennedy J, Eberhart R, editors. Particle swarm optimization. Neural Networks, 1995 Proceedings, IEEE International Conference on; 1995 Nov/Dec 1995.

[30] Bratton D, Kennedy J, editors. Defining a Standard for Particle Swarm Optimization. 2007 IEEE Swarm Intelligence Symposium; 2007 1–5 April 2007.

[31] Kennedy J, Eberhart RC, editors. A discrete binary version of the particle swarm algorithm. Systems, Man, and Cybernetics, 1997 Computational Cybernetics and Simulation, 1997 IEEE International Conference on; 1997 12–15 Oct 1997.

[32] Youden WJ. Index for rating diagnostic tests. Cancer. 1950;3(1):32–5. Epub 1950/01/01. 10.1002/1097-0142(1950)3:1<32::aid-cncr2820030106>3.0.co;2-3 . 15405679

[33] Biggerstaff BJ. Comparing diagnostic tests: a simple graphic using likelihood ratios. Stat Med. 2000;19(5):649–63. Epub 2000/03/04. . 10700737 10.1002/(sici)1097-0258(20000315)19:5<649::aid-sim371>3.0.co;2-h

[34] Meindl A. Identification of Novel Susceptibility Genes for Breast Cancer—Genome-Wide Association Studies or Evaluation of Candidate Genes? Breast Care (Basel). 2009;4(2):93–9. Epub 2009/01/01. 10.1159/000211991 21049069 PMC2931067

[35] Thomas G, Jacobs KB, Kraft P, Yeager M, Wacholder S, Cox DG, et al A multistage genome-wide association study in breast cancer identifies two new risk alleles at 1p11.2 and 14q24.1 (RAD51L1). Nat Genet. 2009;41(5):579–84. Epub 2009/03/31. 10.1038/ng.353 19330030 PMC2928646

[36] Kraft P, Haiman CA. GWAS identifies a common breast cancer risk allele among BRCA1 carriers. Nat Genet. 2010;42(10):819–20. Epub 2010/09/30. 10.1038/ng1010-819 . 20877320

[37] Li J, Humphreys K, Darabi H, Rosin G, Hannelius U, Heikkinen T, et al A genome-wide association scan on estrogen receptor-negative breast cancer. Breast Cancer Res. 2010;12(6):R93 Epub 2010/11/11. 10.1186/bcr2772 21062454 PMC3046434

[38] Yu JC, Hsiung CN, Hsu HM, Bao BY, Chen ST, Hsu GC, et al Genetic variation in the genome-wide predicted estrogen response element-related sequences is associated with breast cancer development. Breast Cancer Res. 2011;13(1):R13 Epub 2011/02/02. 10.1186/bcr2821 21281495 PMC3109581

[39] Fanale D, Amodeo V, Corsini LR, Rizzo S, Bazan V, Russo A. Breast cancer genome-wide association studies: there is strength in numbers. Oncogene. 2012;31(17):2121–8. Epub 2011/10/15. 10.1038/onc.2011.408 . 21996731

[40] Su WH, Yao Shugart Y, Chang KP, Tsang NM, Tse KP, Chang YS. How genome-wide SNP-SNP interactions relate to nasopharyngeal carcinoma susceptibility. PLoS One. 2013;8(12):e83034 Epub 2014/01/01. 10.1371/journal.pone.0083034 24376627 PMC3871583

[41] Greliche N, Germain M, Lambert JC, Cohen W, Bertrand M, Dupuis AM, et al A genome-wide search for common SNP x SNP interactions on the risk of venous thrombosis. BMC Med Genet. 2013;14:36 Epub 2013/03/21. 10.1186/1471-2350-14-36 23509962 PMC3607886

[42] Li P, Guo M, Wang C, Liu X, Zou Q. An overview of SNP interactions in genome-wide association studies. Brief Funct Genomics. 2015;14(2):143–55. Epub 2014/09/23. 10.1093/bfgp/elu036 . 25241224

[43] Chuang LY, Chang HW, Lin MC, Yang CH. Improved branch and bound algorithm for detecting SNP-SNP interactions in breast cancer. J Clin Bioinforma. 2013;3(1):4 Epub 2013/02/16. 10.1186/2043-9113-3-4 23410245 PMC3626712

[44] Chen JB, Chuang LY, Lin YD, Liou CW, Lin TK, Lee WC, et al Preventive SNP-SNP interactions in the mitochondrial displacement loop (D-loop) from chronic dialysis patients. Mitochondrion. 2013;13(6):698–704. Epub 2013/02/19. 10.1016/j.mito.2013.01.013 . 23416325

[45] Ritchie MD, Hahn LW, Roodi N, Bailey LR, Dupont WD, Parl FF, et al Multifactor-dimensionality reduction reveals high-order interactions among estrogen-metabolism genes in sporadic breast cancer. Am J Hum Genet. 2001;69(1):138–47. Epub 2001/06/19. 10.1086/321276 11404819 PMC1226028

[46] Nelson MR, Kardia SL, Ferrell RE, Sing CF. A combinatorial partitioning method to identify multilocus genotypic partitions that predict quantitative trait variation. Genome Res. 2001;11(3):458–70. Epub 2001/03/07. 10.1101/gr.172901 11230170 PMC311041

[47] Ritchie MD, White BC, Parker JS, Hahn LW, Moore JH. Optimization of neural network architecture using genetic programming improves detection and modeling of gene-gene interactions in studies of human diseases. BMC Bioinformatics. 2003;4:28 Epub 2003/07/09. 10.1186/1471-2105-4-28 12846935 PMC183838

[48] Hamon SC, Kardia SL, Boerwinkle E, Liu K, Klos KL, Clark AG, et al Evidence for consistent intragenic and intergenic interactions between SNP effects in the APOA1/C3/A4/A5 gene cluster. Hum Hered. 2006;61(2):87–96. Epub 2006/05/20. 10.1159/000093384 16710093 PMC1698960

[49] McKinney BA, Reif DM, Ritchie MD, Moore JH. Machine learning for detecting gene-gene interactions: a review. Appl Bioinformatics. 2006;5(2):77–88. Epub 2006/05/26. 16722772 10.2165/00822942-200605020-00002 PMC3244050

[50] Musani SK, Shriner D, Liu N, Feng R, Coffey CS, Yi N, et al Detection of gene x gene interactions in genome-wide association studies of human population data. Hum Hered. 2007;63(2):67–84. Epub 2007/02/07. 10.1159/000099179 . 17283436

[51] Moore JH, Asselbergs FW, Williams SM. Bioinformatics challenges for genome-wide association studies. Bioinformatics. 2010;26(4):445–55. Epub 2010/01/08. 10.1093/bioinformatics/btp713 20053841 PMC2820680

[52] Yang P, Ho JW, Yang YH, Zhou BB. Gene-gene interaction filtering with ensemble of filters. BMC Bioinformatics. 2011;12 Suppl 1:S10 Epub 2011/03/05. 10.1186/1471-2105-12-S1-S10 21342539 PMC3044264

[53] Chuang LY, Lin YD, Chang HW, Yang CH. An improved PSO algorithm for generating protective SNP barcodes in breast cancer. PLoS One. 2012;7(5):e37018 Epub 2012/05/25. 10.1371/journal.pone.0037018 22623973 PMC3356401

[54] Tang JY, Chuang LY, Hsi E, Lin YD, Yang CH, Chang HW. Identifying the association rules between clinicopathologic factors and higher survival performance in operation-centric oral cancer patients using the Apriori algorithm. Biomed Res Int. 2013;2013:359634 Epub 2013/08/29. 10.1155/2013/359634 23984353 PMC3741931

[55] Wu SJ, Chuang LY, Lin YD, Ho WH, Chiang FT, Yang CH, et al Particle swarm optimization algorithm for analyzing SNP-SNP interaction of renin-angiotensin system genes against hypertension. Mol Biol Rep. 2013;40(7):4227–33. Epub 2013/05/23. 10.1007/s11033-013-2504-8 . 23695493

[56] Yang CH, Lin YD, Chuang LY, Chen JB, Chang HW. MDR-ER: balancing functions for adjusting the ratio in risk classes and classification errors for imbalanced cases and controls using multifactor-dimensionality reduction. PLoS One. 2013;8(11):e79387 10.1371/journal.pone.0079387 24236125 PMC3827354

[57] Zhang Y, Wu L. Crop classification by forward neural network with adaptive chaotic particle swarm optimization. Sensors (Basel). 2011;11(5):4721–43. Epub 2011/12/14. 10.3390/s110504721 22163872 PMC3231381

[58] Yang CH, Lin YD, Chuang LY, Chang HW. Double-bottom chaotic map particle swarm optimization based on chi-square test to determine gene-gene interactions. Biomed Res Int. 2014;2014:172049 10.1155/2014/172049 24895547 PMC4033510

[59] Ou-Yang F, Lin YD, Chuang LY, Chang HW, Yang CH, Hou MF. The Combinational Polymorphisms of ORAI1 Gene Are Associated with Preventive Models of Breast Cancer in the Taiwanese. Biomed Res Int. 2015;2015:281263 Epub 2015/09/18. 10.1155/2015/281263 26380267 PMC4561876

[60] Lee CH, Ko AM, Warnakulasuriya S, Yin BL, Sunarjo, Zain RB, et al Intercountry prevalences and practices of betel-quid use in south, southeast and eastern Asia regions and associated oral preneoplastic disorders: an international collaborative study by Asian betel-quid consortium of south and east Asia. Int J Cancer. 2011;129(7):1741–51. 10.1002/ijc.25809 . 21128235

[61] Lee CH, Ko AM, Yen CF, Chu KS, Gao YJ, Warnakulasuriya S, et al Betel-quid dependence and oral potentially malignant disorders in six Asian countries. Br J Psychiatry. 2012;201(5):383–91. Epub 2012/09/22. 10.1192/bjp.bp.111.107961 . 22995631

[62] Chiang SL, Chen PH, Lee CH, Ko AM, Lee KW, Lin YC, et al Up-regulation of inflammatory signalings by areca nut extract and role of cyclooxygenase-2 -1195G>a polymorphism reveal risk of oral cancer. Cancer Res. 2008;68(20):8489–98. Epub 2008/10/17. 68/20/8489 [pii] 10.1158/0008-5472.CAN-08-0823 . 18922923

[63] Health Promotion Administration, Ministry of Health and Welfare, Taiwan (R.O.C.). Cancer registration system annual report. 2016.

[64] Chang MC, Ho YS, Lee PH, Chan CP, Lee JJ, Hahn LJ, et al Areca nut extract and arecoline induced the cell cycle arrest but not apoptosis of cultured oral KB epithelial cells: association of glutathione, reactive oxygen species and mitochondrial membrane potential. Carcinogenesis. 2001;22(9):1527–35. 10.1093/carcin/22.9.1527 . 11532876

[65] Chiang SL, Jiang SS, Wang YJ, Chiang HC, Chen PH, Tu HP, et al Characterization of arecoline-induced effects on cytotoxicity in normal human gingival fibroblasts by global gene expression profiling. Toxicol Sci. 2007;100(1):66–74. Epub 2007/08/08. kfm201 [pii] 10.1093/toxsci/kfm201 . 17682004

[66] Ross AC, Zolfaghari R. Cytochrome P450s in the regulation of cellular retinoic acid metabolism. Annu Rev Nutr. 2011;31:65–87. Epub 2011/05/03. 10.1146/annurev-nutr-072610-145127 . 21529158 PMC3789243

[67] Evans TR, Kaye SB. Retinoids: present role and future potential. British journal of cancer. 1999;80(1–2):1–8. Epub 1999/07/02. 10.1038/sj.bjc.6690312 10389969 PMC2362988

[68] Kanojia D, Vaidya MM. 4-nitroquinoline-1-oxide induced experimental oral carcinogenesis. Oral oncology. 2006;42(7):655–67. 10.1016/j.oraloncology.2005.10.013 . 16448841

[69] Moore JH. The ubiquitous nature of epistasis in determining susceptibility to common human diseases. Hum Hered. 2003;56(1–3):73–82. 73735. 10.1159/000073735 . 14614241

[70] Yang CH, Chuang LY, Chen YJ, Tseng HF, Chang HW. Computational analysis of simulated SNP interactions between 26 growth factor-related genes in a breast cancer association study. OMICS. 2011;15(6):399–407. 10.1089/omi.2010.0028 . 21599519

[71] Lin GT, Tseng HF, Yang CH, Hou MF, Chuang LY, Tai HT, et al Combinational polymorphisms of seven CXCL12-related genes are protective against breast cancer in Taiwan. OMICS. 2009;13(2):165–72. 10.1089/omi.2008.0050 . 19196101

[72] Moore JH, Williams SM. New strategies for identifying gene-gene interactions in hypertension. Ann Med. 2002;34(2):88–95. . 12108579 10.1080/07853890252953473

[73] Collins RL, Hu T, Wejse C, Sirugo G, Williams SM, Moore JH. Multifactor dimensionality reduction reveals a three-locus epistatic interaction associated with susceptibility to pulmonary tuberculosis. BioData Min. 2013;6(1):4 10.1186/1756-0381-6-4 23418869 PMC3618340

[74] Oh DY, Jin MH, Lee YS, Ha JJ, Kim BK, Yeo JS, et al Identification of Stearoyl-CoA Desaturase (SCD) Gene Interactions in Korean Native Cattle Based on the Multifactor-dimensionality Reduction Method. Asian-Australas J Anim Sci. 2013;26(9):1218–28. 10.5713/ajas.2013.13058 25049903 PMC4093401

